# Rates, Delays, and Completeness of General Practitioners’ Responses to a Postal Versus Web-Based Survey: A Randomized Trial

**DOI:** 10.2196/jmir.6308

**Published:** 2017-03-22

**Authors:** Paul Sebo, Hubert Maisonneuve, Bernard Cerutti, Jean Pascal Fournier, Nicolas Senn, Dagmar M Haller

**Affiliations:** ^1^ Primary Care Unit Faculty of Medicine University of Geneva Geneva Switzerland; ^2^ Unit of Research and Development in Medical Education Faculty of Medicine University of Geneva Geneva Switzerland; ^3^ Department of General Practice Faculty of Medicine University of Nantes Nantes France; ^4^ Department of Ambulatory Care and Community Medicine University of Lausanne Lausanne Switzerland

**Keywords:** participation rate, response time, completeness, survey methods, primary care

## Abstract

**Background:**

Web-based surveys have become a new and popular method for collecting data, but only a few studies have directly compared postal and Web-based surveys among physicians, and none to our knowledge among general practitioners (GPs).

**Objective:**

Our aim is to compare two modes of survey delivery (postal and Web-based) in terms of participation rates, response times, and completeness of questionnaires in a study assessing GPs’ preventive practices.

**Methods:**

This randomized study was conducted in Western Switzerland (Geneva and Vaud) and in France (Alsace and Pays de la Loire) in 2015. A random selection of community-based GPs (1000 GPs in Switzerland and 2400 GPs in France) were randomly allocated to receive a questionnaire about preventive care activities either by post (n=700 in Switzerland, n=400 in France) or by email (n=300 in Switzerland, n=2000 in France). Reminder messages were sent once in the postal group and twice in the Web-based group. Any GPs practicing only complementary and alternative medicine were excluded from the study.

**Results:**

Among the 3400 contacted GPs, 764 (22.47%, 95% CI 21.07%-23.87%) returned the questionnaire. Compared to the postal group, the participation rate in the Web-based group was more than four times lower (246/2300, 10.70% vs 518/1100, 47.09%, *P*<.001), but median response time was much shorter (1 day vs 1-3 weeks, *P*<.001) and the number of GPs having fully completed the questionnaire was almost twice as high (157/246, 63.8% vs 179/518, 34.6%, *P*<.001).

**Conclusions:**

Web-based surveys offer many advantages such as reduced response time, higher completeness of data, and large cost savings, but our findings suggest that postal surveys can be still considered for GP research. The use of mixed-mode approaches is probably a good strategy to increase GPs’ participation in surveys while reducing costs.

## Introduction

Three main methods of collecting general data were usually used in the past in clinical and epidemiological research (face-to-face interviews, telephone interviews, and postal surveys). They were considered to be more or less equivalent in terms of validity of the data obtained, although postal surveys had the advantage of promoting more truthful responses to sensitive questions, costing less, and requiring fewer staff [1].

More recently, Web-based surveys became a new and popular method for collecting data because they are simple to use, inexpensive (no costs for printing, postage and data entry), less time-consuming (immediate survey delivery, real-time data tracking, and no data entry) and correct (high data quality because a structured format minimizes entry of erroneous or unacceptable data and automatic data transfer minimizes data entry errors) [2-6]. In addition, they offer other advantages, such as flexibility in display design (questions can be revised or removed, new questions can be added according to preliminary results) and almost no limit to the number of respondents [2,4,6,7]. Web-based surveys were developed to study various conditions [8-13] or assess the efficacy of Internet-based programs, for example, in preventing smoking relapse [14]. Several authors compared Web-based and postal surveys in terms of validity and/or reliability, and showed little or no difference between the two methods of data collection [8,10-13].

However, Web-based surveys can give rise to specific concerns about response rates, ethical issues (ie, whether researchers truly can promise anonymity and confidentiality, what constitutes informed consent), and selection bias (in relation to age, socioeconomical and education-related bias in access to the Internet) [2]. In particular, response rates seem clearly lower compared to postal-based surveys both in studies surveying nondoctors (either patients or general population) [5,15,16] and doctors [3,7,17,18]. Only a few studies have directly compared postal and Web-based surveys among physicians, usually using mixed-mode designs (making it more difficult to interpret); none to our knowledge studied this question among general practitioners (GPs). Using these mixed-mode designs, Beebe et al [3] (n=326 physicians) showed a statistically significant difference in response rates between two groups after one reminder (post 57% vs Web 47%), but the difference observed was not significant after switching groups for the second reminder, and McMahon et al [7] (n=181 pediatricians) found that response rates after one reminder were 41% by post and 26% in the Web-based group.

In general, response rates to surveys conducted among doctors are lower than surveys among nondoctors [3,6,19]. In addition, GPs are known to be more difficult to recruit than other doctors [20] and more likely to drop out [3,20,21]. Therefore, the results of studies conducted among physicians other than GPs will not necessarily be similar to studies performed in primary care settings.

The aim of our study was to determine through a randomized design whether Web-based surveys are feasible in primary care by assessing GPs’ participation rates, response times, and completeness of data using two modes of questionnaire delivery: postal and Web-based.

## Methods

### Study Site, Population, and Sample Size

This randomized trial was conducted in Western Switzerland (canton of Geneva and Vaud) and France (Alsace and Pays de la Loire) in 2015, as part of a study of GPs’ preventive practices. Based on previous studies, the expected participation rate was 50% in the postal-based group and 20% in the Web-based group [7,19,22]. Assuming that we wanted to detect a difference in response rate of 10% or more between the two groups, with a power of 80%, and a type I error rate of 5%, a minimum of 408 questionnaires had to be completed in each group (postal and Web-based), which led to a sample size of 816 for the postal group and 2040 for the Web-based group. Taking into account anticipated incomplete questionnaires, both samples were increased by approximately 250, and rounded to 1100 and 2300, respectively.

**Figure 1:**
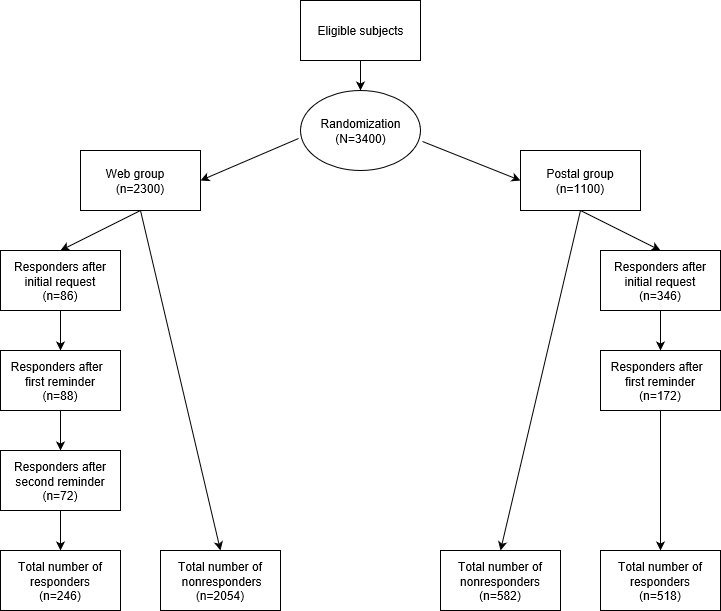
Flowchart of the study.

For this purpose, a random sample of 600 community-based GPs practicing in the canton of Geneva was selected from a sampling frame consisting of all the GPs who were members of the physicians’ professional organization and had a valid and available email address. They were allocated to the postal (n=300) or the Web-based (n=300) group at random, using simple (unrestricted) randomization based on computer-generated random numbers, and invited to participate by post or by email. In addition, 400 GPs were randomly selected in the canton of Vaud. Because a list of email addresses was unavailable (the professional organization of this canton does not make the list available for research purposes), all GPs from the canton of Vaud were allocated to the postal group. Therefore, in Switzerland, 700 GPs were included in the postal group and 300 in the Web-based group. The same procedure—recruitment of community-based GPs by post (n=400) and by email (n=2000)—was carried out in France (Alsace and Pays de la Loire). Reminder messages (once for the postal group and twice for the Web-based group) were sent at one-month intervals. No monetary incentives were offered to the participating GPs. All community-based GPs practicing were eligible for the study, except those practicing only complementary and alternative medicine. There were no other exclusion criteria. This recruitment process is summarized in Figure 1.

### Data Collection

In Switzerland, a research assistant contacted each randomly selected GP by email or by post, according to group allocation. In France, this task was done by the Union Régionale des Professionnels de Santé Alsace and Pays de la Loire. They informed the GPs about the aim of our study and the practical procedures for completing the questionnaire. The postal letters included a cover letter and a stamped return envelope. GPs were asked to send the completed questionnaires back to the research assistant. Participants in the Web-based group received the same cover letter in a Web-based format. GPs were asked to connect through a hyperlink and complete the online questionnaire. The paper questionnaire was designed first, closely following recently published recommendations for optimal survey content and layout [23]. The Web-based questionnaire was then created to be as similar as possible to the paper version, including regarding text formatting. Note that completion of all questions was not required before submission of the Web-based questionnaire.

The questionnaire included questions about sociodemographic characteristics (age, gender, location of the practice, certification, number of working days per week, number of working years in the current practice), as well as about current preventive practices, scored on a five-point Likert scale ranging from “never performed” to “always performed.” There were 37 items in the questionnaire. The analysis of these data will be presented in a separate paper.

The questionnaire was piloted with seven GPs in a primary care clinic (Centre Médical des Trois Chêne, Geneva, Switzerland) to identify any source of difficulties or misunderstanding that colleagues may face when responding to the questions, but only minor changes in the layout were suggested during this pretest phase. All collected data remained confidential. Only the research assistants knew the name and participation code of the GPs taking part in our study; they were not known at any time by the researchers who analyzed the study data. Tacit consent was presumed from the doctors if they completed a questionnaire. We did not collect data about nonresponding GPs. For Switzerland, a waiver from obtaining informed consent was granted by the Research Ethics Committee of Geneva (approval by the Ethics Committee is unnecessary in Switzerland when only physicians are surveyed about their practice), whereas for France the research protocol was approved by the Research Ethics Committee of Nantes (ref: 2015-09-06).

### Statistical Analysis

Comparisons of different categorical variables within contingency tables were made with chi-square tests. Continuous data were summarized by means and standard deviations, and comparisons were made with *t* tests. When the data were clearly asymmetric, medians and interquartile ranges were used, and Wilcoxon rank sum tests for comparisons. The association between the completeness of questionnaires and various variables (gender, age group, country, type of survey, number of half-days worked per week, number of working years) were investigated with generalized linear models with binomial link functions. All significant variables were included in a multivariate model, and the final multivariate model was chosen with a backward and forward stepwise procedure based on the Akaike information criterion [24].

All analyses were run on R 2.15.3 (the R Foundation for Statistical Computing) and TIBCO Spotfire S+ 8.1 for Windows (TIBCO Software Inc).

## Results

### Characteristics and Representability of the Respondents

Among the 3400 GPs who were contacted at random (2400 in France and 1000 in Switzerland), 764 (22.47%) responded to the survey, 336 (14.00%) in France and 428 (42.80%) in Switzerland. Figure 1 shows the flowchart of the study and Table 1 presents the sociodemographic characteristics of the responders according to the group (postal vs Web-based) they were allocated to. There were more men (62.5%, 318/509 vs 49.8%, 110/221, *P*=.002) and GPs older than 55 years (51.6%, 266/516 vs 39.6%, 88/222, *P*=.005) in the postal group compared to the Web-based group. The GPs’ profiles were similar in the two countries (men: 59.1%, 240/406 in Switzerland vs 58.0%, 188/324 in France, *P*=.82; age ≥55 years: 48.9%, 199/407 vs 46.8%, 155/331, *P*<.001).

**Table 1:** General practitioners’ characteristics (N=764).

Characteristics		Web group	Postal group	*P*	Total
**Gender, n/N (%)^a^**				.002	
	Male	110/221 (49.8)	318/509 (62.5)		428/730 (58.6)
	Female	111/221 (50.2)	191/509 (37.5)		302/730 (41.4)
**Age group (years), n/N (%)^a^**				.005	
	<35	16/222 (7.2)	13/516 (2.5)		29/738 (3.9)
	35-44	53/222 (23.9)	104/516 (20.2)		157/738 (21.3)
	45-54	65/222 (29.3)	133/516 (25.8)		198/738 (26.8)
	55-64	75/222 (33.8)	207/516 (40.1)		282/738 (38.2)
	>64	13/222 (5.9)	59/516 (11.4)		72/738 (9.8)
Mean number of half-days worked per week, mean (SD)		8.5 (2.1)	8.6 (2.3)	.80	8.6 (2.2)
Number of working years in the current practice, mean (SD)		16.4 (11.0)	18.7 (11.0)	.01	18.0 (11.1)

^a^ n=number with factor considered; N=number of data available. Denominators do not all add to 764 because of missing values.

For Switzerland, the postal sample appeared to be slightly more representative of all community-based GPs (median age of 56 years and 78% men in 2015 [25]) regarding the median age (median 53.7, IQR 45.7-60.9, for the postal group, and 45.9, IQR 42.9-60.9, for the Web-based group) and gender distribution (men: 215/353, 60.9%, for the postal group and 25/53, 47%, for the Web-based group).

For France (data from Pays de Loire only; median age of 51 years and 57% men in 2013 [26]) the results were similar. The postal sample appeared to be slightly more representative of all community-based GPs regarding the median age (50.9, IQR 41.5-58.7, for the postal group and 47.1, IQR 38.5-56.2, for the Web-based group) and gender distribution (men: 58/97, 60%, for the postal group and 53/114, 46.5%, for the Web-based group).

### Differences in Response Rates Between Groups

Table 2 compares GPs’ participation rates in the two groups. Overall, participation rates were more than four times higher in the postal compared to the Web-based group (47.09%, 518/1100 vs 10.70%, 246/2300, *P*<.001), although the difference was less pronounced in Switzerland (50.7%, 355/700, vs 24.3%, 73/300, *P*<.001) compared to France (40.8%, 163/400 vs 8.65%, 173/2000, *P*<.001). In the Web-based group, the rates of GPs who participated after the initial request, the first, or the second reminder were relatively similar, whereas in the postal group, only half as many GPs completed the questionnaire after the reminder compared to the initial request.

**Table 2:** General practitioners’ participation rates in Web and postal groups stratified by country and by initial request or reminders.

Characteristics		Web group		Postal group		*P*	Total	
		n/N (%)^a^	95% CI	n/N (%)^a^	95% CI		n/N (%)^a^	95% CI
**Switzerland**		73 /300 (24.3)	19.5%-29.2%	355/700 (50.7)	47.0%-54.4%	<.001	428/1000 (42.8)	39.7%-45.9%
	Initial request	21 (7.0)	4.1%-9.9%	245 (35.0)	31.5%-38.5%		266 (26.6)	23.9%-29.3%
	Reminder #1	22 (7.3)	4.4%-10.3%	110 (15.7)	13.0%-18.4%		132 (13.2)	11.1%-15.3%
	Reminder #2	30 (10.0)	6.6%-13.4%	NA	NA		30 (3.0)	1.9%-4.1%
**France**		173/2000 (8.7)	7.4%-9.9%	163/400 (40.8)	35.9%-45.6%	<.001	336/2400 (14.0)	12.6%-15.4%
	Initial request	65 (3.3)	2.5%-4.0%	101 (25.3)	21.0%-29.5%		166 (6.9)	5.9%-7.9%
	Reminder #1	66 (3.5)	2.5%-4.1%	62 (15.5)	12.0%-19.0%		128 (5.3)	4.4%-6.2%
	Reminder #2	42 (2.1)	1.5%-2.7%	NA	NA		42 (1.8)	1.2%-2.3%
**Total**		246/2300 (10.7)	9.4%-12.0%	518/1100 (47.1)	44.4%-50.0%	<.001	764/3400 (22.5)	21.1%-23.9%
	Initial request	86 (3.7)	3.0%-4.5%	346 (31.5)	28.7%-34.2%		432 (12.7)	11.6%-13.8%
	Reminder #1	88 (3.8)	3.0%-4.6%	172 (15.6)	13.5%-17.8%		260 (7.6)	6.8%-8.5%
	Reminder #2	72 (3.1)	2.4%-3.8%	NA	NA		72 (2.1)	1.6%-2.6%

^a^ n=number of GPs agreeing to participate; N=number of GPs contacted in the Web group (Geneva: n=300; Vaud: n=0; Alsace: n=1000; Pays de la Loire: n=1000) and in the postal group (Geneva: n=300; Vaud: n=400; Alsace: n=200; Pays de la Loire: n=200).

### Differences in Response Time Between Groups

In Switzerland, the median response time for paper surveys was approximately 3 weeks for initial request and reminder (Table 3), whereas in France, the response time was 1 week for initial request and 3 weeks for the reminder. In contrast, in both countries, GPs allocated to the Web-based group completed the questionnaire about 1 day after they had received the initial request, the first, or the second reminder.

**Table 3:** General practitioners’ responses times in the two groups stratified by country and by initial request or reminders.

Characteristics		Web group (days), median (IQR)	Postal group (days), median (IQR)	*P*	Total (days), median (IQR)
**Switzerland**		1 (1-3)	21 (16-26)	<.001	19 (14-24)
	Initial request	2 (1-8)	21 (16-27)	<.001	21 (14-24)
	Reminder #1	0 (0-1)	19 (15-25)	<.001	18 (14-25)
	Reminder #2	1 (1-3)	NA	NA	1 (1-3)
**France**		1 (0-2)	10 (6-24)	.78	4 (0-10)
	Initial request	1 (0-2)	6 (6-10)	<.001	5 (1-6)
	Reminder #1	0 (0-3)	25 (20-28)	<.001	6 (0-24)
	Reminder #2	1 (0-2)	NA	NA	1 (0-2)
**Total**		1 (0-3)	20 (14-25)	<.001	14 (2-23)
	Initial request	1 (0-3)	20 (13-23)	<.001	16 (6-23)
	Reminder #1	0 (0-3)	21 (17-28)	<.001	15 (1-25)
	Reminder #2	1 (1-2)	NA	NA	1 (1-2)

### Differences in Questionnaire Completion Between Groups

The number of GPs who fully completed the Web-based questionnaire was nearly 1.5 times higher in Switzerland and more than twice as high in France compared to the completion of postal questionnaires (Table 4). In multivariate analyses, the proportion of fully completed questionnaires was higher for GPs working more (>8 half-days per week), those being less experienced (≤18 years in the current practice), and those having completed the online version of the questionnaire, whereas GP gender and age group, and location of the practice (Switzerland or France) were not associated with full completion of the questionnaire (Table 5).

**Table 4:** Number of general practitioners in the Web and postal groups who fully completed the questionnaire, without missing data, stratified by country.

Characteristics	Web group		Postal group		*P*	Total	
	n/N (%)^a^	95% CI	n/N (%)^a^	95% CI		n/N (%)^a^	95% CI
Switzerland	37/73 (50.7)	39.2%-62.15%	125/355 (35.2)	30.2%-40.2%	<.001	162/428 (37.9)	33.3%-42.4%
France	120/173 (69.4)	62.5%-76.2%	54/163 (31.1)	25.9%-40.3%	<.001	174/336 (51.8)	46.4%-57.1%
Total	157/246 (63.8)	57.8%-69.8%	179/518 (34.6)	30.5%-38.7%	<.001	336/764 (44.0)	40.5%-47.5%

^a^ n=number of GPs having agreed to participate; N=number of GPs contacted.

**Table 5:** Proportion of fully completed questionnaires by sociodemographic characteristics of the responders and type of survey.

Characteristics		n/N (%)	OR (95% CI)	*P*	AOR (95% CI)^a^	*P*
**Gender**				.07		
	Male	185/428 (43.2)	1.00			
	Female	151/302 (50.0)	1.31 (0.98-1.77)			
**Age group (years)**				<.001		
	<35	16/29 (55.2)	1.00			
	35-44	78/157 (49.7)	0.80 (0.31-2.02)			
	45-54	107/198 (54.0)	0.96 (0.38-2.41)			
	55-64	115/282 (40.8)	0.56 (0.23-1.39)			
	>64	20/72 (27.8)	0.31 (0.11-0.90)			
**Number of half-days worked per week**				.005		.007
	≤ 8	127/315 (40.3)	1.00		1.00	
	>8	209/412 (50.7)	1.52 (1.13 2.05)		1.55 (1.12-2.16)	
**Number of working years in the current practice**				.009		.01
	≤18	192/374 (51.3)	1.00		1.00	
	>18	144/346 (41.6)	0.68 (0.50-0.91)		0.66 (0.48-0.91)	
**Location of the practice**				<.001		
	France	174/336 (51.8)	1.00			
	Switzerland	162/428 (37.9)	0.57 (0.42-0.76)			
**Type of survey**				<.001		<.001
	Postal	179/518 (34.6)	1.00		1.00	
	Web	157/246 (63.8)	3.34 (2.43-4.59)		4.42 (3.10-6.30)	

^a^ Adjusted for all variables listed in the table.

## Discussion

### Main Findings

Participation rates were more than four-fold higher when GPs were sent a questionnaire by post than by email. But Web-based questionnaires were completed in a timelier and more complete manner compared to postal questionnaires. The findings were similar in two French-speaking countries.

### Comparison With Previous Studies

#### Response Rates

Previous studies compared participation rates between postal and Web-based surveys among nonphysicians (either general population or patients), and their findings point in the same direction. In a study involving Danish women referred for mammography (N=376), Kongsved et al [15] showed that the response rates were much higher for the postal (73%) compared to the Web-based survey (18%); the questionnaire consisted of 17 pages with a total of 119 items. Another study by Bergeson et al [5] assessed patient experiences with care in Minnesota (N=1392) and found a response rate of 33% by post (vs 14% for a Web-based survey). Finally, Sinclair et al [16] carried out a community survey of greywater use (N=1621) and showed a 10.5% and 7.5% response rate for a personalized and for a generic postal survey, vs 4.7% and 2.2% for a personalized and for a generic postal invitation with Web survey. The low participation rates in this study are probably explained by the study population (general population) and the absence of reminders.

Only a few studies addressed this issue among physicians, usually using mixed-mode designs (making it more difficult to interpret), and none to our knowledge among GPs. These studies provide findings similar to our own results. Beebe et al [3] recruited 326 physicians from 12 divisions within the Mayo Clinic Department of Medicine and showed a statistically significant difference in response rate between the two groups after one reminder (post 57% vs Web 47%), but the difference observed was not significant after switching groups for the second reminder (mail/Web group, that is mail invitation, mail reminder #1, Web reminder #2: 71%; Web/mail group: 63%, *P*=.07). According to the authors, the relatively high response rates in this study could be explained by the fact that Mayo Clinic physicians are particularly interested in research. In a survey that recruited 181 pediatricians in Georgia (USA), McMahon et al [7] found that response rates after one reminder were 41% by post and 26% in the Web-based group. Lower participation rates (26% in the postal vs 11% in the Web-based group) were noted by Hardigan and al [17] among practicing dentists (N=1232) in the Florida Tobacco Control Survey. Finally, in a survey on attitudes toward the screening and treatment of hepatitis C (N=398), Kroth and al [18] showed that, despite a total of five rounds of online solicitation, 24% of the responses were in paper form (the paper version was mailed only to the nonrespondents to the online solicitation).

We found that sending reminders improved response rates in both groups. However, in the Web-based group, the percentage of GPs agreeing to participate after the initial request and the reminders was relatively similar, whereas in the postal group, approximately half as many GPs completed the questionnaire following a reminder (vs the initial request). This finding means that it is probably useful in Web-based surveys to send two or even more reminders, which can be done without extra costs, bearing in mind that too many reminders may be considered as possible harassment of potential respondents [20]. In contrast, the cost-effectiveness of reminders can be unfavorable in postal surveys because costs are constant whereas participation rates tend to decrease for reminders. In addition to increasing the number of reminders, the following strategies have been shown to favor higher response rates in Web-based surveys: attaching a copy of the questionnaire to the email invitation, recruiting physicians with prior experience with Web surveys, using mixed-mode designs (Web/mail or mail/Web), and allowing participants to respond in the desired format (Web or mail) using personalized invitations and financial incentives [3,6,20,27,28].

An important limitation with Web-based surveys is related to the fact that the physicians’ email addresses are neither always available nor regularly updated, which happens less frequently for postal addresses because the physicians have to use them in their current practice for contact with the authorities, other health care providers, and their patients.

#### Response Time

We found that the median response time was significantly longer for postal compared to Web-based surveys; therefore, if a short response time is required, a Web-based survey could be more appropriate than a postal survey. Our results are only in part explained by the fact that postal surveys require several days to be sent and returned, whereas Web-based surveys and responses are delivered immediately. Indeed, the much longer delay for postal responses suggests a longer delay both in opening the invitation letter and in sending back responses. Our results are consistent with previous research; in Beebe et al’s study [3], the median response time was approximatively two days shorter in the Web/postal compared to the postal/Web group, but the results are somewhat difficult to analyze because the two groups were mixed. In McMahon et al’s study [7], 23% of the Web-based questionnaires were returned on the same day versus none in the postal survey. Finally, Akl et al [29] randomized 119 residents in a university-based internal medicine residency program and showed a shorter mean response time (by 3.8 days) in a Web-based survey compared to a postal survey.

#### Completeness of Questionnaires

We showed that the number of GPs who fully completed the questionnaire was higher in the Web-based group compared to the postal group. Although both questionnaire formats were designed to be similar, this suggests that completion of a Web-based questionnaire is less subject to attention errors than a paper questionnaire. Our data are supported by Kongsved et al’s study [15] in which 98% filled in a complete questionnaire in the Web group versus 35% in the mail group, and by McMahon et al’s study [7] in which 2.1% of the questions were not answered in the mail group versus only 0.4% in the Web group. Interestingly, in the multivariate analysis, the proportion of fully completed questionnaires was higher for GPs working more days in the practice and for those who were less experienced, who may be more interested in research because they were involved in clinical research in their recent residency program. To our knowledge, this study is the first to describe questionnaire completeness by sociodemographic characteristics of the respondents. Because completeness of data are as important as response rates, researchers should probably take these results into consideration when conducting questionnaire studies among GPs.

#### Profile of Responders

The GPs in the postal group were a little more representative of the whole community than the Web-based group. The finding that older GPs were less prone to participate in the Web survey is well-known [2]. Interestingly, despite the conventional tendency to view the Web as rather male dominated, we also showed that women were more likely than men to participate in the Web survey; this is probably related to the demographic shift in GP populations with a higher proportion of female GPs in the younger age groups (<35 years: 72% women vs 28% men; >64 years: 15% women vs 85% men). This is also in line with recent studies that tend to show that a growing number of women take part in online research and that, with regard to gender, the so-called “digital divide” tends to disappear [4]. These findings also highlight the fact that in primary care a Web-based survey is more likely to introduce selection bias than a postal survey.

### Limitations

First, only GPs with an available email address (with the exception of GPs practicing in the canton of Vaud) and those practicing in Western Switzerland and two regions in France were invited to participate; this sample could not be representative of all GPs practicing in French-speaking parts of Europe. In addition, email addresses are not regularly updated (which is less the case for postal addresses because the doctors have to use them regularly in their current practice), which could lead to a decrease in participation rates because doctors with incorrect email address may not have received the request to take part in the study. Second, the sampling procedure led to having groups that were considerably different in size. In addition, group allocation cannot be considered to be completely at random because all GPs from the canton of Vaud were allocated to the postal group due to unavailability of email addresses; this might have introduced selection bias. However, the multivariate analysis suggested this bias to be minimal because patterns of responses depended more on the format of the survey and the experience in years and level of activity of the GPs than on the location of activity. Third, only 764 GPs took part in the survey when 816 were expected. The targeted sample size could not be reached in the Web-based group, although the objective was reached for the postal group. Therefore, the study was slightly underpowered. Fourth, although the theme of our study could theoretically have an influence on participation rates, we did not think that this was the case because our results were comparable with many previous studies on various topics and populations. Fifth, the Web- and the paper-based questionnaires were similar regarding text formatting. However, we cannot entirely exclude some degree of measurement error related to minor differences between the two versions of the questionnaire. For example, responders to the four-paged paper version had to turn pages and continue on the back of the sheet, which was not the case for responders of the Web version. However, analysis of the pattern of missing responses showed that there were not significantly more missing responses for questions on the back page of the paper questionnaire compared to the Web version. Finally, we applied recommended strategies to improve response rates in the development of the paper questionnaire. The Web-based questionnaire was then created to be very similar to the paper version, but the extent to which the design of the Web-based version was equally optimal to favor response is unknown.

In conclusion, Web-based surveys lead to reduced response times, higher completeness of data, and cost savings, but postal surveys can still be considered for studies involving GPs to limit low response rates and selection bias. Mixed-mode approaches (postal and Web-based surveys) are probably a good strategy to increase GPs’ participation in surveys while reducing costs. Researchers can use these mixed-mode designs in two ways: they can allow respondents to respond to the form that is the most appropriate for them or they can use a two-step strategy, including an initial postal survey followed by a Web-based reminder to nonrespondents or vice versa.

## Abbreviations

GP: general practitioner
